# Recognition of Negative Emotion Using Long Short-Term Memory with Bio-Signal Feature Compression

**DOI:** 10.3390/s20020573

**Published:** 2020-01-20

**Authors:** JeeEun Lee, Sun K. Yoo

**Affiliations:** 1 Graduate Program of Biomedical Engineering, Yonsei University, Seoul 03722, Korea; jeunlee@yuhs.ac; 2 Department of Medical Engineering, Yonsei University College of Medicine, Seoul 03722, Korea

**Keywords:** emotion, bio-signal, auto-encoder, LSTM

## Abstract

Negative emotion is one reason why stress causes negative feedback. Therefore, many studies are being done to recognize negative emotions. However, emotion is difficult to classify because it is subjective and difficult to quantify. Moreover, emotion changes over time and is affected by mood. Therefore, we measured electrocardiogram (ECG), skin temperature (ST), and galvanic skin response (GSR) to detect objective indicators. We also compressed the features associated with emotion using a stacked auto-encoder (SAE). Finally, the compressed features and time information were used in training through long short-term memory (LSTM). As a result, the proposed LSTM used with the feature compression model showed the highest accuracy (99.4%) for recognizing negative emotions. The results of the suggested model were 11.3% higher than with a neural network (NN) and 5.6% higher than with SAE.

## 1. Introduction

Emotion occurs through a complex interaction of stimuli and is used as an indicator to infer one’s psychological and emotional state [1]. In particular, negative emotion is a highly awakening state that appears in such psychological states as anxiety, fear, and anger. Negative emotion causes stress and lowers attention and concentration [2]. Recognizing a negative emotion is the starting point for addressing risk factors. From this perspective, it is very important to classify negative emotions.

A variety of methods including questionnaire evaluation interviews, facial expressions, and gestures are used to discriminate emotions [3]. These techniques reflect personal thinking, culture, age, and gender and can result in manipulation [4,5,6,7]. In contrast, bio-signals do not allow any intentional manipulation, although they present one’s personal psychological state according to stimuli. Accordingly, if a bio-signal is used to discriminate emotions, it is possible to obtain more objective and more accurate information than with personal responses.

Bio-signals result from the responses of the central nervous system and the autonomic nervous system, which change according to external stimulation. The bio-signals that are used to discriminate emotions include electroencephalogram (EEG), electrocardiogram (ECG), skin temperature (ST), and galvanic skin response (GSR) [4]. Among these bio-signals, EEG is a signal that represents the response of the central nervous system and is often used to discriminate emotions. There were many studies where there was excellent performance when an EEG single signal was used to discriminate arousal and valence [8,9,10]. However, it is inconvenient to attach EEG electrodes. Unlike for EEG, signals of the autonomic nervous system (such as ECG, ST, and GSR) require only simple attachment of electrodes. In fact, emotion generates complex biological responses; therefore, there have been studies on complex bio-signals, including the signals of the autonomic nervous system, in order to discriminate emotions [11,12,13].

By analyzing the time domain, frequency domain, and statistical analysis of bio-signals, it is possible to extract various features [5]. Due to the fine current flow of a bio-signal that is measured, the signal is sensitive to movement, external noise, etc. [14]. If complex bio-signals are utilized, the feature vector of a high dimension can lower classifier performance or cause over-fitting [15]. Given that, it is important to select a bio-signal feature that fits the discrimination of emotions.

To increase an emotion classifier’s performance, it is important to design a classifier and to select features appropriately. For the classification of emotions, a variety of classifiers, such as support vector machines (SVMs), Bayesian networks, correlation analysis, Fisher linear discriminant projection, and fuzzy interference, are utilized. These classifiers have an accuracy in the range of 75% to 98%. In particular, when a Bayesian network is trained using EEG, the accuracy is 98%. On the other hand, when an SVM is trained using the autonomic nervous system, the accuracy is 74% [8,16,17,18,19]. To discriminate equivocal emotions, it is important to analyze the emotional mood induced over time. In addition, nonlinear operation is needed to analyze complex reactions. These days, deep learning based on nonlinear operations shows excellent performance in diverse fields. By applying it to emotion classification, it is possible to design an emotion classifier with high performance.

To discriminate negative emotions, this study utilized complex bio-signals. In particular, a stacked auto-encoder (SAE) was utilized to compress bio-signal features. Additionally, long short-term memory (LSTM), which shows excellent performance in the analysis of time series data, was applied to the design of a new emotion recognizer.

## 2. Methods

### 2.1. Bio-Signal Acquisition

In this study, two types of videos were used to generate emotions. A test taker watched a 60-min documentary video that was used to induce neutral emotions. After adequate rest, the test taker was asked to watch a 60-min horror video, which was used to provoke negative emotion. While the test taker was watching a video, bio-signal data were obtained. The test takers were nineteen men in their 20s who had no psychological or physical disorders, and who signed a consent form before the tests. Using 1 kHz sampling with a MP 150TM of BIOPAC Systems, Inc., CA, USA, multiple signals (ECG, ST, and GSR) were measured. Before a test, a test taker was instructed not to move during the test in order to minimize the sensor noise generated by movement [20].

### 2.2. Bio-Signal Feature

#### 2.2.1. Feature Extraction

A sliding window that moved thirty seconds per five minutes was applied to the bio-signals measured to extract a feature vector. An ECG is a signal that reflects activity of the autonomic nervous system. If negative emotions are provoked, the heart beats rapidly and thereby R-peak intervals become narrower [21]. To extract a feature from an ECG, the Pan–Tompkins algorithm was used to detect R-peaks [22]. Based on the detected R-peaks, heart rate variability (HRV) was calculated, and its time domain and frequency domain were analyzed for feature extraction. The features extracted in the analysis on the time domain of the HRV included the mean of R-peak intervals (mean HRV), the standard deviation of R-peak intervals (SDNN), the square root of the mean squared difference of successive R-peaks (RMSSD), the number of pairs of successive R-peaks that differ by more than 50 ms (NN50), and the proportion derived by dividing NN50 by the total number of R-peak intervals (pNN50). The features extracted from the frequency domain included the ratio of low-frequency power and high-frequency power (LF/HF), total frequency power (TF) in the 0.003–0.4 Hz range, normalized high-frequency power (nHF) in the range 0.15–0.4 Hz, and the normalized low-frequency power (nLF) in the range 0.04–0.15 Hz [20,23].

In the case of ST, the speed of a response to a stimulus is fast. To remove the noise from the obtained ST, 50 Hz down-sampling and then low pass filtering was applied. The features extracted from ST included the mean skin temperature (mean ST) and the standard deviation of skin temperature (SD ST) [20,24].

A GSR signal was used as the scale to find the level of activity of the sympathetic nervous system. If an emotion changes a lot, a GSR signal has substantial vibration. For this reason, it is possible to use the signal as the main feature for emotion analysis. GSR consists of a phasic component that represents skin conductance response (SCR), and a tonic component that represents the skin conductance level (SCL). A phasic component is one that vibrates and changes rapidly according to stimuli, and a tonic component is a level of in vitro activity of sweat glands [25]. Discrete wavelet transform (DWT) was conducted to separate the phasic from the tonic components and to extract features. The features extracted from the separated phasic component included the zero-crossing of galvanic skin response for the phasic feature (ZC GSRP) and the standard deviation of the galvanic skin response for the phasic feature (SD GSRP). The features extracted from the tonic component included the mean galvanic skin response for the tonic feature (mean GSRT), the standard deviation of galvanic skin response for the tonic feature (SD GSRT), and the amplitude of galvanic skin response for the tonic feature (Amp GSRT) [20].

#### 2.2.2. Feature Vector Processing

In this study, it was assumed that it is hard to generate an emotion and catch the emotional mood induced during the introductory part of a video. For this reason, the signals of 100 window segments extracted in the latter half of a video were applied. The features extracted from one window segment are presented in Table 1. There were sixteen features (nine of ECG, two of ST, and five of GSR). In the basic emotions per test taker, there were 100 x 16 feature vectors, and in the negative emotions, there were 100 x 16 feature vectors. Therefore, a total of 200 x 16 feature vectors was generated.

The obtained bio-signals included noise associated with movement. Outliers occurred in features with noise. The outliers were recovered using linear interpolation. The feature values of bio-signals were extracted from such different domains as time domain and frequency domain. If a feature vector was applied without normalization, a value range could be different depending on features and such a difference could influence the weight. Therefore, each feature value was normalized between 0 and 1 using a z-score based on the mean and standard deviation.

### 2.3. Emotion Recognizer 

#### 2.3.1. Feature Compression

Individuals express different degrees of emotion. Therefore, if an emotion is not induced, the measured signal looks like noise. If a variety of feature vectors are used, it is necessary to remove duplicated features and select bio-signal features associated with negative emotions in order to improve the performance of the emotion recognizer. An auto-encoder (AE) trains a weight vector for input reconstruction; accordingly, the weight learned by an AE is used for such processes as denoising and data compression [26].

This study utilized a stacked auto-encoder (SAE) for feature compression. First, an AE with sixteen hidden nodes was learned so as to reconstruct sixteen features. Next, an AE with eight hidden nodes was learned that compressed sixteen hidden nodes. Last, the weight values of two learned AEs were added together to make the SAE. Equation (1) represents loss ($L_{SAE}$) of SAE, where N is the number of samples, 𝕩 is the feature vector, and ${𝕩}^{\prime }$ represents a feature vector. $L_{SAE}$ was calculated by adding the mean square error (MSE), L2 regularization ($L_{2}$), and all the sparsity loss (ρ). In particular, sparsity loss, which is generated in a hidden node, constrains activation so as to have the weight be learned randomly and to implement an independent type of feature representation [27].

(1) $$L_{SAE}=\frac{1}{N}\sum \left(𝕩-{𝕩}^{\prime }\right)+\;L_{2}+\rho$$

#### 2.3.2. Emotion Recognizer

Emotion changes over time; moreover, the bio-signals obtained from an induced emotion are also sequential. In addition, emotion reflects the constant mood generated by visual stimuli, the influence of the states felt before the stimuli, and other complex factors. Therefore, an emotion recognizer was designed with the use of LSTM, which shows excellent performance in terms of the analysis of time series data.

When using various features as input, the performance of the classifier can be improved. However, when using duplicated or irrelevant features, the classifier becomes overfitted or its performance is lowered. Therefore, dimension reduction of a feature is more important for improving classifier performance rather than using direct features. Therefore, the second hidden node of SAE is used as input. In this study, bi-directional LSTM with twenty hidden units was applied for bi-directional information learning. The learning by LSTM is shown in Equations (2)–(4), where F is the compressed feature vector extracted by SAE, 𝕨 is the weight vector of the hidden units, and b is the bias. The term 𝕪 t is the final output and is classified in neutral and feature states using the softmax layer. The newly designed LSTM is learned in the direction of reducing an error through an Adam optimizer and cross-entropy [28].

(2) 𝕪t=softmax𝕨𝕙𝕪 concat𝕙t→,𝕙t←+b𝕪,

(3) $$\vec{{𝕙}_{t}}=\tanh\left({𝕨}_{{𝕩}^{\prime }\vec{𝕙}}F_{t}+\;{𝕨}_{\vec{𝕙}\vec{𝕙}}{\vec{𝕙}}_{t-1}+\;b_{\vec{𝕙}}\right)$$

(4) $$\vec{{𝕙}_{t}}=\tanh\left({𝕨}_{{𝕩}^{\prime }\vec{𝕙}}F_{t}+\;{𝕨}_{\vec{𝕙}\vec{𝕙}}{\vec{𝕙}}_{t-1}+\;b_{\vec{𝕙}}\right)$$

**Figure 1:**
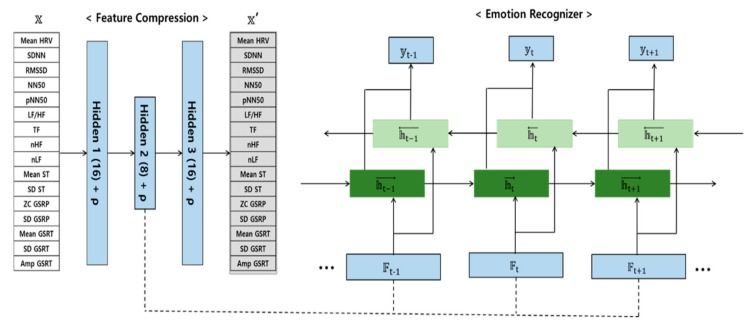
Architecture of the emotion recognizer.

Figure 1 illustrates the overall architecture of the emotion recognizer utilized in this study. The emotion recognizer consisted of SAE and LSTM. The eight features extracted from SAE were used as input to the LSTM. Using the compressed features, the LSTM learned the emotion classifier. The leave-one-out cross-validation (LOOCV) method was used for model validation. LOOCV is useful for small datasets [29,30]. Since the total number of datasets was 19, 18 of the datasets were used for training and one of the datasets was used for testing. The process was repeated 19 times by changing the test set.

## 3. Results

### 3.1. Extracted Features

**Figure 2:**
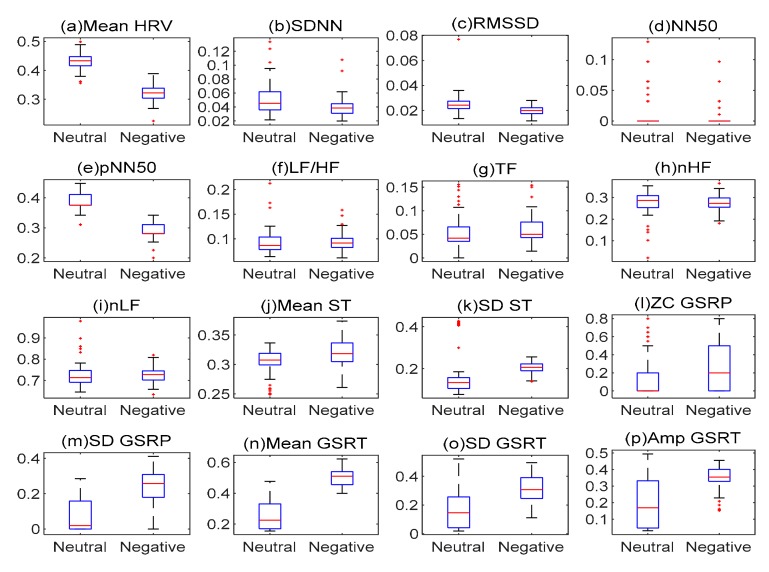
Scatter plot of extracted features: (**a**) mean HRV, (**b**) SDNN, (**c**) RMSSD, (**d**) NN50, (**e**) pNN50, (**f**) LF/HF, (**g**) TF, (**h**) nHF, (**i**) nLF, (**j**) mean ST, (**k**) SD ST, (**l**) ZC GSRP, (**m**) SD GSRP, (**n**) mean GSRT, (**o**) SD GSRT, and (**p**) Amp GSRT.

Figure 2 illustrates a scatter plot of the features extracted in the event that neutral emotion and negative emotion were induced, showing the mean and standard deviation according to each feature. In terms of the mean and standard deviation, the mean HRV, SDNN, RMSSD, and pNN50 extracted from ECG, the SD ST extracted from TF and ST, and the features extracted from GSR were different depending on emotions. In contrast, others (NN50, LF/HF, nHF, nLF, and mean ST) showed no big difference in the mean and standard deviation in relation to emotions. This result revealed that the features extracted from bio-signals were valid for recognition of emotion. Since the extracted features included duplicated ones and invalid features for each emotion, it was necessary to select different features depending on specific emotions.

### 3.2. Results of Auto-Encoding

**Figure 3:**
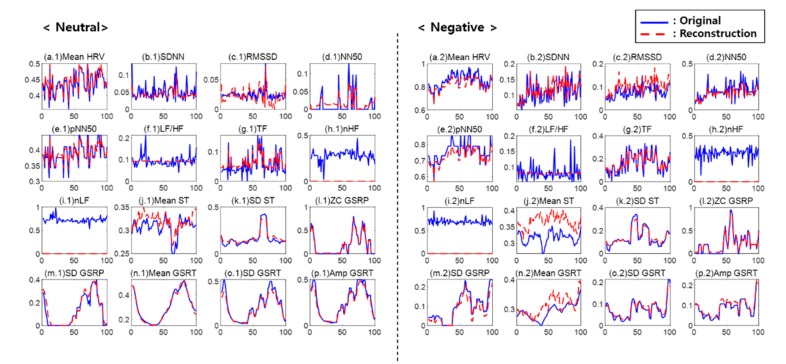
Reconstruction result of the first auto-encoder: (**a**) mean HRV, (**b**) SDNN, (**c**) RMSSD, (**d**) NN50, (**e**) pNN50, (**f**) LF/HF, (**g**) TF, (**h**) nHF, (**i**) nLF, (**j**) mean ST, (**k**) SD ST, (**l**) ZC GSRP, (**m**) SD GSRP, (**n**) mean GSRT, (**o**) SD GSRT, and (**p**) Amp GSRT.

Figure 3 illustrates each one of the features reconstructed using the first AE with sixteen hidden nodes. In the figure, the blue solid line indicates an original value, and the red dotted line indicates a reconstructed feature value. The left side is a neutral emotion value, and the right side is a negative emotion value. The first AE played a role in removing the noise of outliers (de-noising) with the uses of features extracted from the ECG (mean HRV, SDNN, RMSSD, NN50, pNN50, and TF), from ST, and from GSR. It was also found that the features influencing a classifier and causing noise, such as LF/HF, nHF, and nLF, were removed and reconstructed.

**Figure 4:**
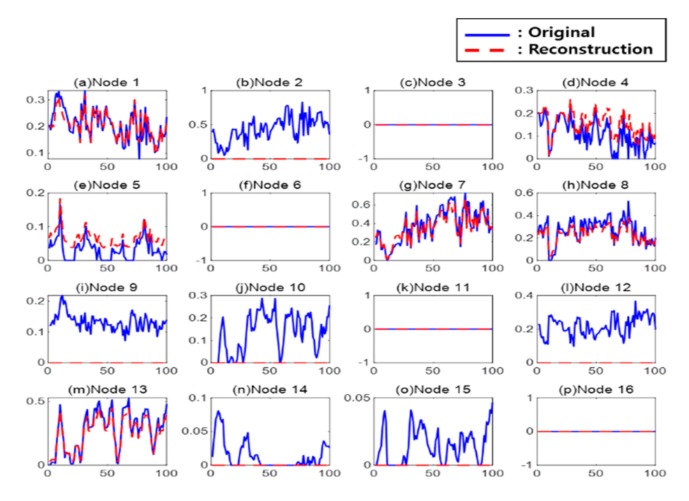
Reconstruction result of second auto-encoder: (**a**) Node 1, (**b**) Node 2, (**c**) Node 3, (**d**) Node 4, (**e**) Node 5, (**f**) Node 6, (**g**) Node 7, (**h**) Node 8, (**i**) Node 9, (**j**) Node 10, (**k**) Node 11, (**l**) Node 12, (**m**) Node 13, (**n**) Node 14, (**o**) Node 15, and (**p**) Node 16.

Figure 4 shows the results from the reconstruction of the second AE with eight hidden nodes. The second AE reconstructed features using the hidden nodes of hidden layer 1 as input. In the figure, the blue solid line represents a hidden node of hidden layer 1, and the red dotted line represents a reconstructed hidden node of hidden layer 3. In the first AE, the 3rd, 6th, 11th, and 16th nodes were inactive. Through the second AE, the 2nd, 9th, 10th, 12th, 14th, and 15th nodes of hidden layer 3 were inactive. As a result, the features became compressed.

**Figure 5:**
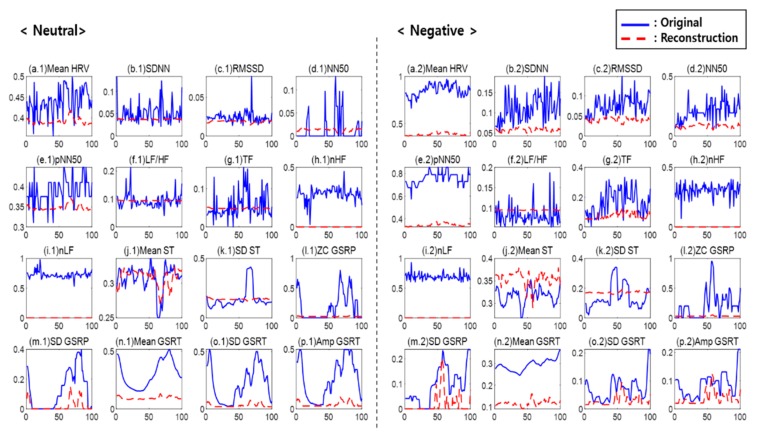
Reconstruction result of stacked auto-encoder: (**a**) mean HRV, (**b**) SDNN, (**c**) RMSSD, (**d**) NN50, (**e**) pNN50, (**f**) LF/HF, (**g**) TF, (**h**) nHF, (**i**) nLF, (**j**) mean ST, (**k**) SD ST, (**l**) ZC GSRP, (**m**) SD GSRP, (**n**) mean GSRT, (**o**) SD GSRT, and (**p**) Amp GSRT.

Figure 5 illustrates the reconstruction result of two stacked AEs (SAE). In the figure, the blue solid line represents an original value, and the red dotted line represents a reconstructed feature value. The left side is a neutral emotion value, and the right side is a negative emotion value. Through SAE, a reconstructed feature value became lower, but the feature trend remained unchanged. The ECG features (mean HRV, RMSSD, NN50, pNN50, and TF, ST’s mean ST, and GSR’s SD GSRP, mean GSRT, SD GSRT, and Amp GSRT) were reconstructed even after the SAE, whereas the values of SDNN, LF/HF, nHF, nLF, SD ST, and ZC GSRP almost converged. Through the SAE, the nodes of each hidden layer were inactivated, thereby compressing the valid features of emotion recognition.

### 3.3. Classification Performance

Table 2 shows the mean and standard deviation of performance comparison of the proposed classifier developed by combining the SAE and LSTM, a neural network (NN), a deep neural network (DNN), a deep belief network (DBN), and the SAE. The NN and DNN had no feature compression functions. The DBN and SAE were learned in the NN-based fine-tuning process after feature compression. The performance comparison was conducted between classifiers in the event that features were not compressed, and in the event that the time information was not applied.

Some of the parameters of each model were fixed. The batch size was five, the initial learning rate was reduced from 0.01, and the max epoch was fixed from 5000 to convergence. Other parameters, which were the number of hidden layers and the number of hidden nodes, were designed by changing the optimum parameters. NN consisted of one hidden layer with 16 hidden nodes and was trained through the sigmoid activation function. The DNN had four hidden layers and each hidden layer had 64, 64, 32, and 16 hidden nodes. The activation function of the hidden layer was rectified linear unit (ReLU), and the activation function of the output layer was softmax. The DBN had two hidden layers. Each hidden layer consisted of 16 and 8 hidden nodes. Each hidden layer was trained through a restricted Boltzmann machine (RBM). The hidden layer was trained again through fine-tuning with two output nodes. SAE was composed by stacking softmax layers with two nodes instead of LSTM proposed in this study. The software for implementation of the classifiers was Neural Network Toolbox 11.1 provided by Matlab 2019 of MathWorks, Inc., Boston, USA.

The SAE–LSTM combined classifier had the highest accuracy (98.4%). In addition, its sensitivity and specificity were also the highest (96.7 and 100%, respectively). In contrast, when the simple NN was used, its accuracy was the lowest (87.4%) and the difference between its sensitivity and specificity was > 6%. The accuracy of DNN was 91.3%, about 3.5% higher than the accuracy of the NN. The difference between the sensitivity and specificity was not large. The DBN and SAE played a role in compressing the features through unsupervised learning. Both of the two classifiers had similar accuracy rates (94.4 and 95.2%, respectively), which were the next highest after the newly proposed classifier.

In this study, statistical evaluation was done to confirm the significance of the suggested model. The Wilcoxon signed ranks test was used because it evaluates the significance of each method in the same subject. Table 3 shows the statistical evaluation results of the Wilcoxon signed rank test. The model combined with SAE and LSTM, which was suggested, was significant compared to other classifiers (*p* < 0.01).

## 4. Discussion

In this study, bio-signals that provide objective information were obtained to enable recognition of negative emotions. Through the SAE, the valid emotion features extracted from bio-signals were compressed, and the compressed features (along with time information) were learned by LSTM.

Emotion is so subjective that it is difficult to quantify a degree of emotion in evaluations. For this reason, these researchers measured neutral emotions and negative emotions so as to find how different the degree of emotion was between individuals. To overcome the problem of quantification, such bio-signals as ECG, ST, and GSR are used as indicators of biological responses. If negative emotions are induced, one’s heart rate beats faster, and R-peak intervals become narrower [21]. As shown in Figure 2a (mean HRV), the mean HRV value was lower when negative emotion was induced than when neutral emotion was induced. In addition, as presented in Figure 2k (SD ST), the ST change was larger when negative emotion was induced than when neutral emotion was induced. In Figure 2n (mean GSRT), negative emotion caused a high level of sweat gland activity.

Bio-signals are sensitive to noise like that associated with movement [16]. Therefore, to improve an emotion classifier’s performance, it is necessary to perform denoising and feature-compression [26]. AE plays a role in removing noise, inactivating a hidden node, and thereby reducing a dimension. In this study, the first AE removed outlier values generated by movement (etc.), so as to do denoising. The result of reconstruction also revealed that the values of LF/HF, nHF, and nLF converged to zero. As shown in Figure 2, these features had no big difference in terms of the mean of each emotion so that they were not valid for classification. The second AE reconstructed the hidden nodes of the first AE and compressed the features. In this way, it also played a role in inactivating the six hidden nodes of the third hidden layer so as to extract the features significant for emotion classification. The reconstruction result of the final SAE (two stacked AEs) revealed that LF/HF, nLF, nHF, SDST, and ZC GSRP were not significant for emotion discrimination. In contrast, the features mean HRV, SD GSRP, mean GSRT, SD GSRT, and Amp GSRT remained unchanged after reconstruction. Accordingly, it is possible to infer that these features play a critical role in classifying emotions. The hidden nodes of the second hidden layer were compressed features and were used as input of a classifier.

A variety of classifiers, including NN and SVM, were applied to design an emotion recognizer [8,16]. In fact, emotion changes over time and is influenced by the moods before and after the emotion is provoked. For this reason, to improve the performance of an emotion classifier, it is necessary to take into consideration changes that occur in time series. The bi-directional LSTM proposed in this study takes into account bi-directional time, and it has better performance than do other models such as NN and DNN. The DBN and SAE, which are learned through unsupervised learning, show better performance than do NN and DNN; however, both DBN and SAE (which combine NN after feature compression), fail to reflect time-series characteristics. Therefore, the SAE–LSTM combined model proposed in this study has the best performance in comparison with the other classifiers. The proposed model has a standard deviation of about 4%. Emotion is affected by factors such as personal thinking, which results in differences in performance according to individuals. SAE selects features that are sensitive to emotion through feature compression, and LSTM detects changes over time. These factors can analyze individual-independent effects, and thus the proposed model can reduce the influence of individuals.

## 5. Conclusions

For the discrimination of negative emotions in this study, associated features were extracted from bio-signals, and a time series-based classifier was designed. The SAE was applied to compress features. With the compressed features, LSTM was learned. In this case, a negative emotion classifier had 99.4% accuracy. The performance of this emotion classifier was 5.6% higher than that of the emotion classifier that used SAE only. Since the SAE–LSTM combined model extracted valid features and reflected the time-series characteristics, it showed better performance than the other classifiers did. There are open databases such as the Emotional Movie Database (EMDB) and Database for Emotion Analysis using Physiological Signals (DEAP) for emotion recognition from biological signals [31,32]. However, there is a limit to using data obtained from a limited experimental environment. In addition, the proposed model has a variety of parameters and the parameters can be changed. Therefore, it is necessary to verify the generalization performance of the model for the state-of-the-art datasets acquired through new experiments later and to optimize the model factors. Discriminating negative emotions is the starting point to resolving the risk factors derived from depression, etc. Therefore, it could be applicable to diverse areas including clinical treatment and the development of emotion products.

## Figures and Tables

**Table 1:** Features extracted from the bio-signals.

Signal	Extracted Features
ECG	Mean HRV, SDNN RMSSD, NN50, pNN50, LF/HF, TF, nHF, nLF
ST	Mean ST, SD ST
GSR	ZC GSRP, SD GSRP, Mean GSRT, SD GSRT, Amp GSRT

ECG (electrocardiogram); ST (skin temperature); GSR (galvanic skin response); HRV (heart rate variability); SDNN (standard deviation of R-peak intervals); RMSSD (square root of the mean squared difference of successive R-peaks); LF/HF (low-frequency power and high-frequency power); TF (total frequency power); SD ST (standard deviation of skin temperature); ZC GSRP (zero-crossing of galvanic skin response for the phasic feature); SD GSRP (standard deviation of the galvanic skin response for the phasic feature); GSRT (galvanic skin response for the tonic feature); nHF (normalized high-frequency power); nLF (normalized low-frequency power).

**Table 2:** Performance according to classifiers.

Value	Accuracy	Sensitivity	Specificity
Suggested	98.4 ± 3.7	96.7 ± 3.7	100.0 ± 0.0
NN	87.8 ± 3.9	91.1 ± 5.0	84.4 ± 5.6
DNN	91.3 ± 3.0	89.1 ± 4.8	93.2 ± 3.9
DBN	94.4 ± 3.2	95.5 ± 2.9	93.0 ± 5.6
SAE	95.2 ± 5.9	95.3 ± 7.3	95.8 ± 5.9

**Table 3:** Statistical evaluation using Wilcoxon rank sum test.

Value	W	z	*p*-Value
NN	536	4.9	*P* < 0.01
DNN	527	4.7	*P* < 0.01
DBN	500	3.9	*P* < 0.01
SAE	464	2.8	*P* < 0.01
